# Personalized virotherapy in cancer

**DOI:** 10.18632/aging.100750

**Published:** 2015-05-18

**Authors:** Eduardo G Cafferata, Osvaldo L Podhajcer

**Affiliations:** Laboratory of Molecular and Cellular Therapy, Fundacion Instituto Leloir, IIBBA-CONICET, Argentina

After decades of intensive research, pancreatic adenocarcinoma (PDAC) remains a highly lethal disease with a median survival time that does not exceed 6.5 months [1]. Since more than 80% of patients present with advanced metastatic disease, systemic chemotherapy remains the only treatment. The high resistance to conventional and targeted therapies in PDAC as in other desmoplastic tumors of adults is largely due to the dense extracellular matrix (ECM). The ECM distorts blood and lymphatic vessels structure that hampers the possibility of systemically delivered therapies to reach the tumor mass [2].

The use of engineered oncolytic viruses (OVs) is a promising new therapy for cancer treatment. Different OVs have been engineered to express immune stimulatory molecules indicating that OVs can act at two levels, by directly killing malignant cells in concurrence with the simultaneous activation of the host anti-tumor immunity. OVs can be also combined with chemotherapeutic agents providing an aggressive platform for cancer attack. One of these OVs, a Herpes Simplex virus named T-VEC armed with GM-CSF has just completed a phase III trial in advanced melanoma with promising results and might reach the clinic after FDA approval (Clinical http://Trials.gov Identifier NCT01740297).

Conditionally Replicative Adenoviruses are oncolytic adenoviruses (OAV) engineered to selectively replicate within and kill tumor cells. Selectivity is obtained through the use of “cancer cell”- specific promoters (CCSP) that are selected to replace viral promoters and drive the expression of genes essential for OAV replication. OAVs replicate essentially in malignant cells with positive expression of the gene from which the CCSP was selected. OAVs efficacy can be also improved through the exchange of the capsid fiber of the virus or addition of specific moieties that will retarget vectors to enter the cell through alternative receptors [3].

As for other therapeutic modalities, viral spread and therapeutic efficacy is hampered by the ECM barrier. With this in mind, we started engineering OAVs whose replication was driven by CCSPs active both in the stroma and in the malignant compartment of the tumor mass. The OAV AdF512 retargeted to bind to an alternative cell surface receptor and carrying DNA elements responsive to hypoxia and inflammation exhibited enhanced efficacy on melanoma xenografts made of human melanoma cells mixed with human fibroblasts [4]. AdF512v1 was also able to lyse fresh samples obtained from ovary cancer patients including samples refractory to chemotherapy [5]. More recently we have shown that the OAV AV25CDC combined with gemcitabine exhibited a large efficacy and complete absence of toxicity in preclinical models of pancreatic cancer in mice and syriam hamsters [6]. AV25CDC was able to disrupt tumor architecture by inducing an increase in MMP-9 activity that would have facilitated gemcitabine penetration deeply inside the tumor mass [6]. Recent studies have shown that cancer-associated fibroblasts can render malignant cells more permissive to OVs infection through a cross-talk signaling that involves stromal and tumor derived factors [7].

In addition to the efforts to improve viral dissemination and the secondary immune response, it is tempting to anticipate, as with current targeted therapies that not all patients will benefit from this oncolytic immune-virotherapy. Thus, the question arises whether there is any possibility to predict patient response to this treatment. The use of patient derived xenografts (PDXs) is gaining momentum as a tool to predict response [8]. In PDXs modeling, samples obtained from patient's tumors or circulating tumor cells are implanted in immunocompromised mice either ectopically or orthotopically. Once tumors grow in mice different treatment combinations can be implemented to establish the most efficacious one. From available data with other therapeutic modalities it appears that PDX models are the best preclinical model to test different combinations of OAVs with drugs of current use in pancreatic cancer. In addition, genomic firms can be obtained from interrogating resistant vs sensitive PDX tumors. An important caveat in PDAC is that nearly half of original tumors grow in mice, generally within two to six months which in some cases is coincidental with patient survival.

We propose that in order to advance the field of targeted virotherapy it will be important to establish a procedure involving an initial step consisting in the assessment in the patient's tumor biopsy of the expression levels of the viral receptor and the gene corresponding to the CCSP; these data should be complemented with the in vitro lytic effect of the OAV on the tumor explant; in parallel, the therapeutic efficacy of the OAV combined with other treatments should be assessed in the PDXs. The integration of the whole data might help predict virus efficacy combined with other modalities to guide patients’ treatment. These approaches combined with the development of a genomic firm associated with sensitivity vs resistance to virus activity in the PDXs (and in patients) might help in selecting patients in advance that will benefit from OAV therapeutics.
